# The method of educational assessment affects children’s neural processing and performance: behavioural and fMRI Evidence

**DOI:** 10.1038/s41539-017-0010-9

**Published:** 2017-08-29

**Authors:** Steven J. Howard, Hana Burianová, Alysha Calleia, Samuel Fynes-Clinton, Lisa Kervin, Sahar Bokosmaty

**Affiliations:** 1 Early Start and School of Education, Faculty of Social Sciences, University of Wollongong, Wollongong, NSW 2522 Australia; 2 Centre for Advanced Imaging, University of Queensland, Brisbane, QLD 4072 Australia; 3 Psychology Department, Swansea University, Swansea, UK

## Abstract

Standardised educational assessments are now widespread, yet their development has given comparatively more consideration to what to assess than how to optimally assess students’ competencies. Existing evidence from behavioural studies with children and neuroscience studies with adults suggest that the method of assessment may affect neural processing and performance, but current evidence remains limited. To investigate the impact of assessment methods on neural processing and performance in young children, we used functional magnetic resonance imaging to identify and quantify the neural correlates during performance across a range of current approaches to standardised spelling assessment. Results indicated that children’s test performance declined as the cognitive load of assessment method increased. Activation of neural nodes associated with working memory further suggests that this performance decline may be a consequence of a higher cognitive load, rather than the complexity of the content. These findings provide insights into principles of assessment (re)design, to ensure assessment results are an accurate reflection of students’ true levels of competency.

## Educational neuroscience: Method of educational assessment matters

Each year, millions of students sit standardised educational tests; what they assess is similar, how they assess is not – affecting results. An interdisciplinary team led by Dr Steven Howard of Australia’s University of Wollongong monitored patterns of brain activity as 7–8 year old children undertook different forms of standardised assessment. Despite equivalent content in the tests, the methods of assessment engaged working memory networks differentially–with students’ performance declining the more these networks were engaged. These results show that educational assessments must consider not only what to assess, but how to optimally capture students’ emerging knowledge, skills, and abilities. Clear pre-identification of target abilities, followed by experimental evaluation of methods of assessment, can help ensure assessment results accurately reflect students’ true competencies.

## Introduction

National and international programs of standardised educational assessment are now widespread. Countries such as the United Kingdom, United States of America, Canada, Australia, China, Japan, Korea, and Singapore currently administer large-scale standardised educational assessment programs at one or multiple points in a student’s school career for the purpose of providing accurate, meaningful, and actionable indices of students’ knowledge, skills, and abilities.^1–3^ Development of these assessments has involved extensive consideration of the content areas assessed–those that are of national priority, in alignment with curricular expectations, or are predictive of later life outcomes. For instance, Australia’s National Assessment Program–Literacy and Numeracy (NAPLAN), which is administered annually to students at four points in their schooling, was designed to assess the “sorts of skills that are essential for every child to progress through school and life, such as reading, writing, spelling and numeracy.”^4^ In support of this assertion, there is ample evidence that subsequent academic and life outcomes (e.g., high school completion, employment, health, wealth, criminality, and mental wellbeing) are strongly predicted by literacy and numeracy domains commonly assessed by these tests.^5–8^

While there has been clear and explicit consideration of the content that is assessed in standardised educational assessments, there has been comparatively less consideration of how to optimally assess and index students’ competencies in these areas. A major consequence is discrepancy in the methods of assessing the same knowledge, skills, and abilities. Australia’s NAPLAN, for instance, assesses students’ spelling competencies by test-takers identifying and correcting misspelled words.^4^ In contrast, England’s National Curriculum Assessments (often called ‘SATs’) similarly aim to assess students’ spelling abilities, but do so by having students spell the missing word in a sentence after its verbal presentation.^9^ This diversity in the methods of assessment is paralleled throughout the world, across core content domains, in both public and commercial tests.

There is mounting evidence that how content is assessed can have a significant impact on students’ ability to demonstrate their current levels of competency, even after controlling for the complexity of the content. For instance, Willet and Gardiner^10^ found that students correctly spelled more words when presented verbally than when correcting written spelling mistakes. Whether multiple-choice items effectively measure students’ academic abilities has similarly been questioned, as scores are inflated with guessing.^11^ This behavioural evidence, however, is constrained by the possibility that these findings are spurious (due to situational factors, such as fluctuations in motivation) or transitory (e.g., due to practice effects).

Another potential explanation for the discrepant levels of performance across assessments is that different methods of assessment engage different cognitive processes. Within the area of educational assessment, emerging neuroscience research with adults found proofreading (i.e., correct the spelling mistake) and dictation methods of spelling assessment (i.e., spell the verbally presented word) differed in the extent to which they engaged working memory (WM) brain networks.^12^ To explain this finding, it was suggested that the proofreading method of assessment may recruit additional WM resources in evaluating plausible, but incorrect and interfering, letter sequences.^12^ There is also parallel literature to suggest that differing forms of assessment indeed mobilize different underlying cognitive processes, such as in old/new recognition memory vs. forced choice recognition memory tests.^13, 14^ While it is not possible or preferable to create an educational assessment that does not engage WM, due to its well-established indirect effect on learning,^15, 16^ unintended measurement error is introduced when WM also directly influences test performance beyond what is required for demonstrating the target learning. It becomes something that the test *tests*, beyond the construct(s) of interest (e.g., spelling, reading comprehension, or numeracy). This introduces a test impurity issue that could influence test-taker performance in a non-uniform manner along a gradient of students’ WM capacities. This is consistent with core propositions of Cognitive Load Theory, which suggest that the mode of information delivery can influence demands placed on test-takers’ working memory.^17, 18^ Given global inequality in selection of assessment practices to assess content knowledge, this suggests that contemporary approaches to educational assessment may be engaging disparate cognitive processes that unduly influence student performance.

Despite this evidence, every year millions of students undertake NAPLAN assessments in Australia, SATs in the UK, and EQAO in Ontario, among others. There are similar national assessment programs throughout the world. While each test purports to measure comparable knowledge, skills, and abilities (e.g., spelling, grammar, numeracy, or reading), they do so in vastly different ways. Although it seem intuitive that different methods of assessment should engage different cognitive processes (with implications for the accuracy with which different methods index the construct of interest), and while there is indeed parallel literature affirming this with other sorts of tests and tasks (e.g., recognition memory tests),^13, 14^ policymakers and test developers seem to have not acted upon those findings.

The current study therefore sought to provide more direct evidence related to the target of inquiry (e.g., a sample of current assessment practices) and sample of interest (e.g., school-aged students), to answer questions of *how* to assess (not just *what* to assess) students who actually take these tests. Specifically, we used functional magnetic resonance imaging (fMRI) with Australian Grade 2 students (age 7–8 years) to identify and quantify the domain-general contributions to performance on proofreading (i.e., correction of a written spelling error), cloze dictation (i.e., spelling after verbal presentation), and multiple-choice assessments that were otherwise equated in difficulty. Measurement of brain activity was supplemented by in-scanner test performance (an advance over previous research that used out-of-scanner testing to assess the in-scanner performance^12^) to further investigate the relationship between brain and behaviour. This convergence of behavioural and neuroanatomical evidence is important for reconciling emerging brain-based insights (e.g., brain-based evidence of varied cognitive load across different forms of assessment) with current theory to support, refine, or advance established principles of educational best practice.^12, 19^ Not only does the combination of these approaches address a key limitation of behavioural studies–conflation of spurious, transitory, and core processing differences–but it also avoids the pitfalls of defining the mechanisms of learning and performance in purely operationist terms (as psychometric constructs that are measured exclusively by tests) that often are not founded upon theory or understandings of the brain.^19^

In line with the proposal that the method of assessment imposes differing and extraneous cognitive demands, it was expected that: (a) children’s spelling performance would decrease with the increasing cognitive load of assessment (such that proofreading would impose the highest cognitive load and multiple choice the lowest cognitive load); and (b) methods of testing involving higher cognitive load would additionally recruit areas of the frontoparietal network that are associated with working memory and increased attention (e.g., prefrontal and parietal cortices).^20, 21^ To evaluate these hypotheses, behavioural analyses and associated neural correlates are reported.

## Results

### Behavioural spelling performance

To evaluate effects of condition on children’s spelling performance, a repeated-measures ANOVA was conducted on the accuracy scores for each condition. Despite the constrained sample, which limits the ability to detect potentially genuine differences across conditions, results indicated a large main effect of Condition, *F*(2, 26) = 47.11, *p*  < .001, *η* ^2^ = .78. As expected, post-hoc analyses indicated that accuracy was greatest in the multiple-choice condition (*M * = 0.74, *SD*  = 0.20), followed by the cloze dictation condition (*M * = 0.57, *SD * = 0.28), and then the proofreading condition (*M*  = 0.46, *SD*  = 0.29). As expected, correlations between the conditions were high, but non-perfect (ranging from *r * = .87 to .91), suggesting that although the conditions captured a common core of spelling, there remained systematic differences in performance as a function of test condition. While these results were consistent with a priori hypotheses, such that test performance increased with decreasing WM demands, they were further evaluated using fMRI data (given sample size constraints that limit stability of these estimates and our ability to conduct significance tests between correlations).

### fMRI results

**Fig. 1:**
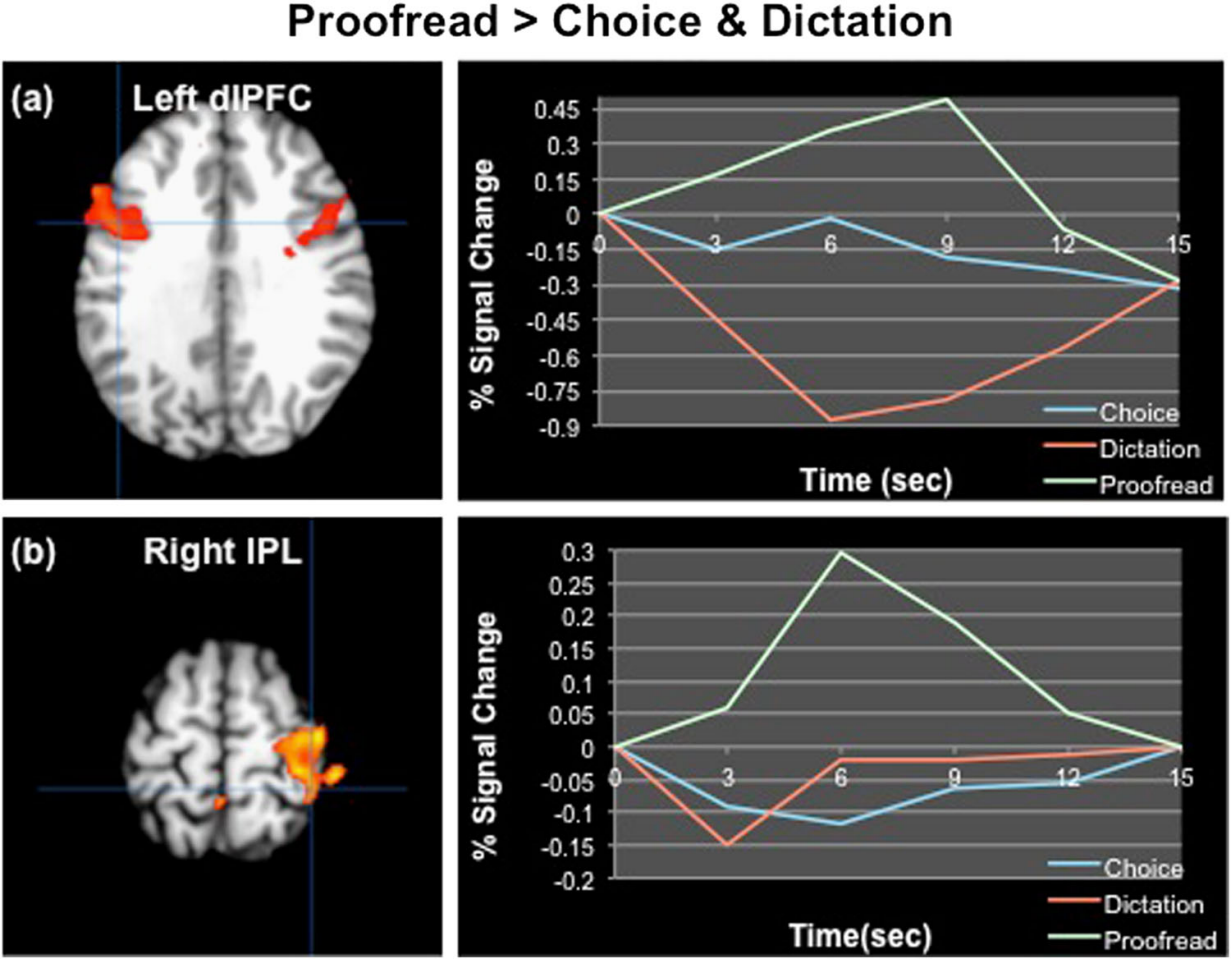
A brain pattern differentiating the proofreading condition from the cloze dictation and multiple-choice conditions during the mental search. **a** The time course of activity in the left dorsolateral prefrontal cortex. **b** The time course of activity in the right inferior parietal lobule. *IPL*  inferior parietal lobule, *dlPFC * dorsolateral prefrontal cortex, *Choice*   multiple-choice condition, *Dictation*   cloze dictation condition, *Proofread*   proofreading condition

**Fig. 2:**
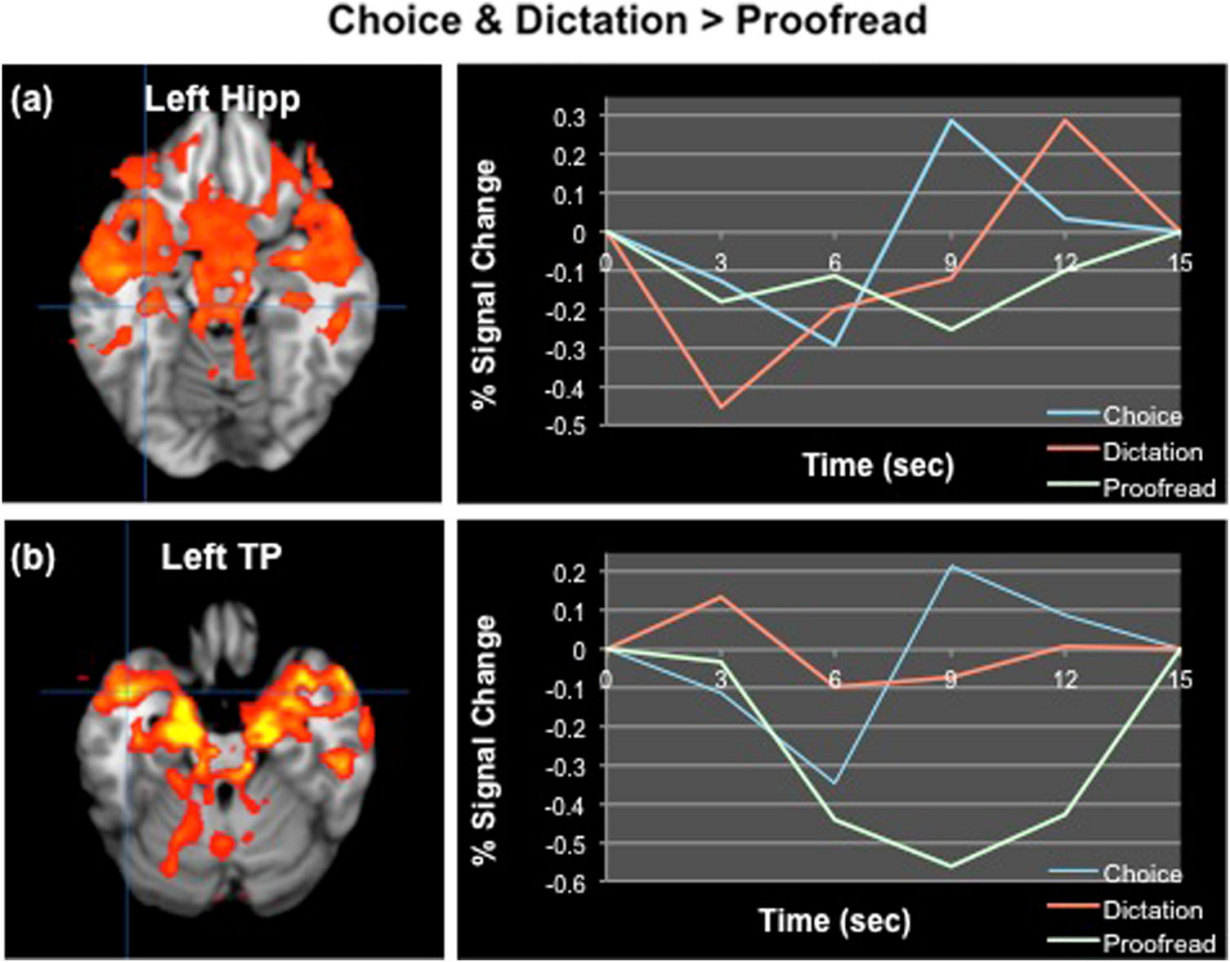
A brain pattern differentiating the multiple-choice and cloze dictation conditions from the proofreading condition during the mental search. **a** The time course of activity in the left hippocampus. **b** The time course of activity in the left temporal pole. *Hipp*   hippocampus, *TP* temporal pole, *Choice*   multiple-choice condition, *Dictation*  cloze dictation condition, *Proofread*   proofreading condition

To assess the neural correlates of the experimental conditions, two separate analyses were conducted, comparing brain activation during the mental search and spelling phases of each condition. During the mental search phase, two statistically significant (*p* < 0.003) patterns of brain activity were identified. The first pattern differentiated the proofreading condition from both the cloze dictation and multiple-choice conditions, accounting for 69.94% of covariance in the data. During the proofreading condition, significantly higher activations were found in bilateral dorsal frontoparietal network (comprising dorsolateral prefrontal cortex and inferior parietal lobule), precuneus, and bilateral fusiform gyrus (see Fig. 1). In contrast, during the cloze dictation and multiple-choice conditions significantly higher activations were found in the bilateral parahippocampus and hippocampus, temporal poles, insula, inferior frontal gyrus, thalamus, basal ganglia, middle and superior temporal gyrus, and left angular gyrus (see Fig. 2). In contrast to the cloze dictation and multiple-choice conditions, which engaged areas important for semantic processing, memory recognition, conceptual integration, and cue-stimulus binding,^22^ the proofreading condition reflected a greater cognitive load and higher attentive control, engaging nodes of the dorsal attentional stream and working memory areas.^20, 21^

The second identified brain pattern differentiated cloze dictation from the multiple-choice condition, accounting for 38.06% of covariance in the data. Multiple choice engaged bilateral middle temporal gyrus, temporal poles, hippocampus, parahippocampus, thalamus, putamen, and inferior frontal gyrus, reflecting the engagement of areas that have been shown to be active during response inhibition, processing of semantic verbal information, accessing of word meaning during reading, and binding of highly processed perceptual inputs.^23, 24^ In contrast, cloze dictation engaged bilateral lingual gyrus, fusiform gyrus, caudate nucleus, medial frontal gyrus, and anterior cingulate cortex, reflecting the monitoring of verbal fluency and identification and recognition of words.^25–27^

**Fig. 3:**
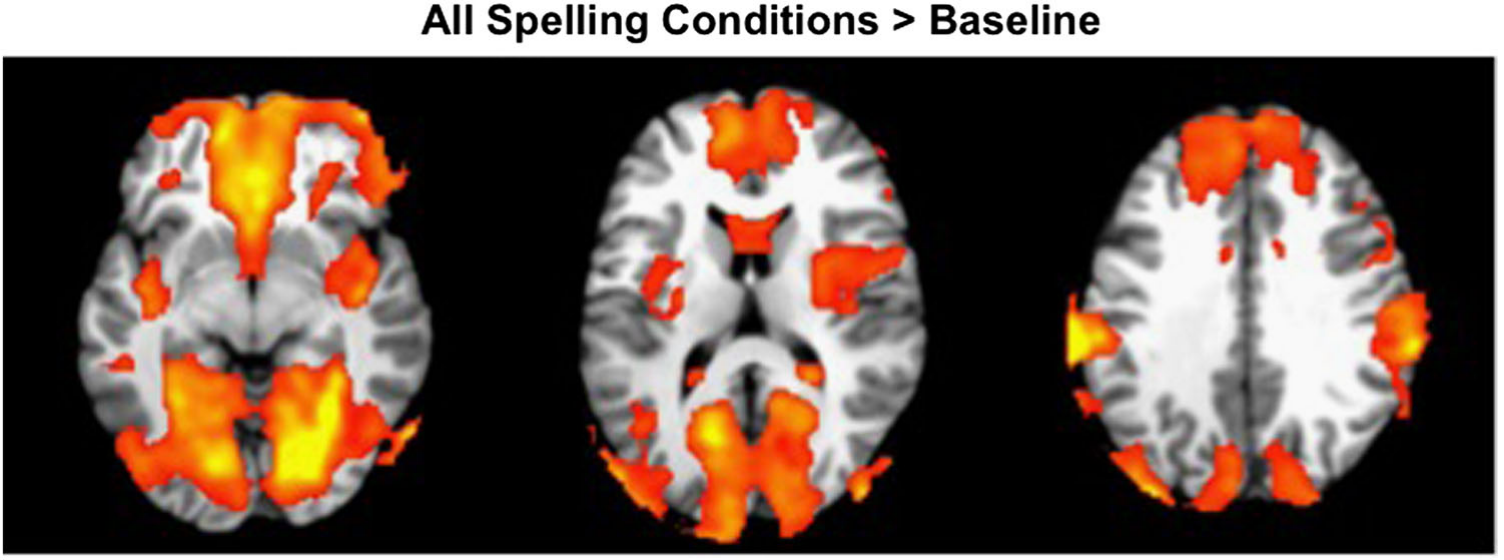
A brain pattern differentiating all spelling conditions from baseline during the mental search

During the spelling phase, all conditions activated a common brain pattern, which comprised the anterior and posterior cingulate gyri, bilateral inferior parietal lobule (angular and supramarginal gyri), precuneus, insula, parahippocampus, hippocampus, fusiform gyrus, inferior frontal gyrus, and lingual gyrus, accounting for 70.91% of covariance in the data (see Fig. 3).

## Discussion

The current study sought to investigate the effect of assessment method on test-takers’ processing and performance, to extend prior behavioural and adult findings suggesting that different methods of assessment may engage fundamentally different cognitive processes. The study also extended those prior investigations to a broader range of assessment methods than have been previously considered. Results indicated that children’s test performance declined as the cognitive load of assessment method increased. Activation of neural nodes associated with WM during performance of the proofreading condition further suggests that the performance decline may be a consequence of additional cognitive load, rather than the complexity of the content (which was equated across spelling lists). In contrast, during the two conditions that imposed comparatively less WM demand, children more highly activated areas associated with verbal fluency and recognition of words during cloze dictation, or areas related to inhibition and memory recognition during multiple choice. These results illustrate, at both the brain and behavioural levels, the effect that different assessment methods have on children’s processing and performance.

Specifically, consistent with our hypotheses and with previous studies,^10, 12, 28, 29^ children’s behavioural results indicated declining performance with increasing WM demands, even after controlling for the complexity of the content. This finding is consistent with previous child and adult findings,^10, 12^ which suggest that proofreading forms of assessment may recruit additional WM resources to activate correct orthographic representations. Specifically, in addition to mental search and spelling processes inherent within cloze dictation spelling (spelling from scratch), proofreading may also involve WM-demanding processes of coordinating grammatical rules to locate the error and then supressing interference from the presented plausible alternative. In contrast, multiple-choice forms of assessment, which yielded the highest performance, may be associated with less WM-demanding recognition processes. Performance on multiple-choice assessments may also be supported by guessing, thereby artificially inflating results.^11^

It could be argued that these assessment approaches *intentionally* differ in the knowledge and skills they aim to assess. For instance, Australia may emphasise spelling in the context of existing print, whereas the UK emphasises the ability to produce spellings. Notable, however, is that educational assessment programs characterise the knowledge and skills they assess in often-identical terms, such as “numeracy”, despite assessing these competencies in a highly disparate manner. As such, another potential explanation for the discrepancy in performance across otherwise equivalent spelling tests is that these different methods of assessment may *unintentionally* impose differing WM demands that are peripheral to the competencies being assessed. This is consistent with core tenets of Cognitive Load Theory, which suggest that information varies in the demands (cognitive load) it places on learners’ WM as a function of its inherent complexity (intrinsic load) and complexity with which information is presented (extraneous load).^17, 18^ For instance, resistance to plausible alternatives, as may be the case in proofreading, may require recruitment of additional attentional resources (a causal factor that underlies WM capacity) to hyper-activate task-relevant information or supress task-irrelevant information.^30, 31^ Although the foremost concern of Cognitive Load Theory has thus far been learning and instructional design, our behavioural and neuroanatomical data suggest that these principles may also apply to the assessment of children’s knowledge and skills (rather than only acquisition of these competencies). This interpretation is compatible with Whelan’s^19^ mapping of fMRI-derived activations to specific sources of cognitive load, which suggests that the current finding of dorsal attentional and WM-related activations during proofreading may have been a consequence of increased intrinsic load. That is, the cognitive processes required for error correction may be intrinsically more complex (higher in element interactivity) than production or recognition of a correct spelling.

It could, of course, be argued that it is not preferable or possible to create an assessment that eliminates WM demands. While correct, previous research showing WM as associated with measurement error in standardised assessments suggests that WM is a dimension that has been directly assessed, even beyond indirect effects of WM on learning and knowledge acquisition.^32^ Instead, it is preferable to maximise variance associated with the competencies being assessed and minimise the variance associated with any extraneous information and processing, as is the case for all test construction. In doing so, WM would only be expected to exert an indirect effect through its central role in learning – or directly when it plays an essential role in the target abilities being assessed (e.g., proofreading requires that students concurrently consider intent, meaning, and language conventions).

It might be argued that this is of minor consequence, as assessment results, ranking, and reactions almost always occur within a program of assessment. Even if a systematic bias does exist, to the extent that an assessment introduces measurement error consistently across test-takers it should preserve relative comparisons across years, regions, schools, and students. Yet the ability to identify student needs and provide tailored educational supports on the basis of these results is a fundamental aim of educational assessment – an aim that necessitates that students’ results accurately reflect their levels of competency. This is well captured by the distinction between ‘assessment of learning’ and ‘assessment for learning’, whereby the latter uses assessment results to provide bespoke educational support and action based on students’ educational progress.^33^ For example, a student who is underperforming on a numeracy test due to literacy or WM constraints, rather than a lack of numeracy knowledge or skills, may not derive benefit from mathematical remediation. There is support for this assertion from findings that WM training can improve numeracy abilities amongst those with low numeracy levels^34^ and children with numeracy-related disabilities who do not improve with remediation tend to show immature WM-related strategies.^35^ Our study thus illustrates the importance of assessment being clearly aligned with, and derived from, the intended learning outcomes.

This study extends previous adult-based neuropsychological investigations and child-based behavioural investigations to show that current methods for assessing domain-specific knowledge and skills differentially affect the processing and performance of test-takers. That is, our results suggest that current approaches to assessment confound non-targeted processes with those that are the target of assessment. This suggestion extends beyond indirect effects of WM on learning, to the demands placed by the assessment type and method. This finding has implications for students’ abilities to learn, demonstrate, and improve their competencies in assessed areas, with follow-on effects for the educational supports they are provided on the basis of their assessment results. Given the prevalence and often high-stakes of standardised educational assessments internationally – such as funding and/or public ranking based on a school’s or region’s results – our findings suggest that development of educational assessments must consider not only what to assess but also how to assess. In this way, assessments can be optimised in their utility as assessments *of* and *for* learning.

## Methods

### Participants

Participants were 14 Australian primary school students in Grade 2 (aged 7–8 years; *M*  = 7.78, *SD =  *0.43; range = 7.09–8.41). This sample size is consistent with comparably designed research that found a robust signal with 12 participants.^12^ Further, the analytical methods that were adopted (i.e., Partial Least Squares (PLS), permutations, bootstrap resampling) are unaffected by small sample size. Bootstrap resampling, in particular, is a distribution-independent method of statistical inference, which is especially recommended when sample size is limited.^36^ Participants were recruited via University newsletters and flyers posted in the community. As a condition for inclusion, participants were healthy, right-handed, and had normal or corrected-to-normal vision and no history of neurological, behavioural, or psychological disorder. One participant was removed from analysis due to excessive motion. In the resultant sample, 72.7% were male (*n*  = 8) and all were native speakers of English. Participants’ parents provided written informed consent, and children gave verbal assent, after a full explanation of the study, in line with the protocols approved by The University of Queensland’s Human Research Ethics Committee.

### Measures

Participants’ spelling abilities were assessed in each of the following three experimental conditions (ordered from highest to lowest working memory demands, per our hypotheses): (1) a *proofreading* condition, in which a sentence contained an unidentified misspelled word to be identified and then corrected (e.g., ‘Sam tryed very hard to study for the test’); (2) a *cloze dictation* condition, in which a sentence contained a missing word to be spelled (e.g., ‘The train ______ at every station’); and (3) a *multiple-choice* condition, in which a sentence contained a missing word with four alternative spellings amongst which to choose (e.g., ‘Millions of _____ visit Sydney each year’: peeple; people; peopel; peepel). Condition (1) was based on Australia’s NAPLAN tests, (2) on the UK’s National Curriculum Tests, and (3) on North American and commercially available standardised spelling assessments. All trials involved textual and auditory presentation of the sentence, after which participants planned a response and then provided this response verbally. The researcher recorded these responses to evaluate accuracy. Each condition consisted of 20 sentences, divided evenly into six runs of 10 sentences each.

Words to be spelled for all conditions were identified as age-appropriate by standardised literacy assessments. Novel sentences were then developed for each of the words and these items were piloted with Grade 2 children (*N*  = 31). Sentences were then divided evenly into the three conditions on the basis of the pilot accuracy rate (*M*_*%*_ = 60.74%, *SD*  = 0.01), word frequency norms (*M*_*freq/500*_ = 110.89, *SD*  = 4.13) grapheme length (*M*_*#letters*_ = 5.70, *SD* = 0.49), phoneme length (*M*_*#sounds*_ = 4.16, *SD* = 0.41), phonetic difference (*M*_*#letters-sounds*_ = 1.54, *SD*  = 0.19), sentence length (*M*_*#words*_ = 8.20, *SD*  = 0.55), and error type. There were no statistically significant differences across sentence lists for these variables (all *p*s < .05). Each condition was administered in pseudo-random order (i.e., presentation order of experimental conditions was randomised; however, conditions were not repeated until each had be presented once), twice per fMRI scan (i.e., 10 sentences per run).

### Procedure

**Fig. 4:**
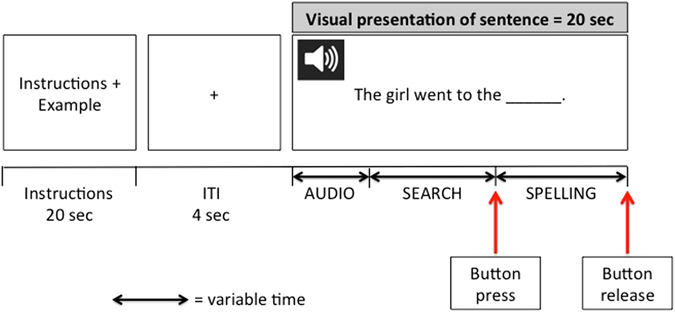
In-scanner experimental paradigm. At the beginning of each run, instructions for the upcoming condition and an example sentence were presented for 20 sec, followed by a 4-sec inter-trial-interval (ITI), and then a 20-sec trial during which a sentence was presented audio-visually. The visual sentence remained on the screen for the entire 20 sec, whereas auditory presentation varied with the length of the sentence (offset of which signalled the start of the search phase). When ready to spell, participants pressed the button (signalling the end of the search phase and start of the spelling phase), releasing it once spelling was completed (signalling the end of the spelling phase)

Accompanied by their parent, participating children completed 10-minute familiarisation training 30 min prior to their scan, which introduced children to the MRI environment, scanning procedures, and task requirements. During subsequent fMRI scanning, children completed six 10-word spelling tests (divided into runs, with each experimental condition being presented twice) over the course of a 45-minute scan. Each run lasted just short of 4.5 min and proceeded as follows: (1) instructions for 20 s, stating condition requirements; (2) fixation for 4 s; (3) visual and auditory presentation of a sentence for 20 s; and (4) repetition of steps 2 and 3 for the run’s remaining 9 sentences. Stimuli were projected onto a screen at the back of the scanner and the participants viewed them through a mirror attached to the head coil. Within each run, the order of sentence stimuli was randomised to eliminate any potential order effects. Participants responded to each trial by: (a) listening to and reading the sentence; (b) mentally preparing a spelling of the target word (mental search phase); (c) pressing and holding a button to indicate the beginning of the spelling phase (during which participants spelled the target word aloud); and (d) releasing the button to indicate completion of spelling (see Fig. 4). This process, automated by each participant during pre-scan familiarisation, allowed for the discrimination of neural activation associated with ‘mental preparation’ of a response (delineated by the sentence’s auditory offset until the participant’s button press) and ‘verbal spelling’ (i.e., provision of a response, delineated by button press and button release). To also consider response accuracy, the researcher recorded participants’ verbal responses manually.

### fMRI data acquisition

Anatomical and functional images were acquired at the Centre for Advanced Imaging, at the University of Queensland, using a 3 Tesla Siemens Magnetom Trio scanner with a 32-channel head coil. Anatomical images were acquired using an MP-RAGE sequence (192 sagittal slices, TR = 1900 ms, TE = 2.32 s, FOV = 230 mm, voxel size = 0.9 mm^3^, TI = 900 ms, flip angle = 9°). Brain activation was assessed using the blood oxygenation level-dependent effect with optimal contrast. Functional images were obtained using a whole head T2*-weighted echo-planar image sequence (85 axial slices TR = 3000 ms, TE = 30 ms, flip angle = 90°, FOV = 192 mm, voxel size = 2.5 mm^3^).

### fMRI data preprocessing & analysis

The fMRI images were preprocessed using Statistical Parametric Mapping software (SPM8; http://www.fil.ion.ucl.ac.uk/spm). Functional images were slice-timing corrected and then realigned onto the mean image for head-motion correction. The anatomical image was then segmented and spatially normalised to the T1-weighted Montreal Neurological Institute template, and normalisation parameters were applied to the functional data. Finally, data were spatially smoothed by convolving each volume with an isotropic Gaussian kernel (FWHM = 6 mm). For analyses, all trials for which participants made a correct response were averaged within and across each condition’s two runs.

The fMRI data were analysed using PLS analysis.^37, 38^ PLS is a multivariate technique that examines the covariance between activity in all brain voxels and experimental conditions, providing sets of mutually independent spatial patterns depicting brain regions that show the strongest relationship to the contrasts across conditions. Using PLS, cohesive patterns of neural activity associated with the task were identified across the three conditions (i.e., proofreading, cloze dictation, and multiple choice). Of primary interest was brain activity during the mental search phase, for which distinct patterns of activation were expected across experimental conditions due to differing processes required to plan a response (whereas the spelling phase should involve identical processes across experimental conditions). We therefore isolated activity during the mental search phase (starting at the offset of auditory presentation of the sentence and ending at onset of spelling, as indicated by a button press) and spelling phase (starting at button press and ending at button release) as distinct events for the event-related analyses. Activity at each time point in the analysis was normalised to activity in the onset time point. The measure of each phase-related activity thus was relatively uninfluenced by activity in the rest of the trial. A permutation test determined significance of each brain pattern and bootstrap estimation of the standard errors determined the reliability of each brain pattern.^39^ Peak voxels with a salience/SE ratio > 3.0 were deemed to be reliable, as this approximates *p * < .003.^40^ Because extraction of the activation patterns is done in a single analytic step, akin to principal component analysis, no correction for multiple comparisons was required.^37, 38^

### Data availability

Data are available from the authors on request.
